# Acute health effects of accidental chlorine gas exposure

**DOI:** 10.1186/s40557-014-0029-9

**Published:** 2014-10-01

**Authors:** Joo-An Kim, Seong-Yong Yoon, Seong-Yong Cho, Jin-Hyun Yu, Hwa-Sung Kim, Gune-Il Lim, Jin-Seok Kim

**Affiliations:** 1 Department of Occupational and Environmental Medicine, Soonchunhyang University Gumi Hospital, Gumi, Gyeongbuk, South Korea; 2 Environmental Health Center, Soonchunhyang University Gumi Hospital, Gumi, Gyeongbuk, South Korea; 3 Department of Preventive Medicine, Soonchunhyang University Medical College, Asan, Chungnam, South Korea; 4 Department of Pulmonology, Soonchunhyang University Gumi Hospital, Gumi, Gyeongbuk, South Korea

**Keywords:** Chlorine, Chemical hazard release, Reactive airway dysfunction syndrome

## Abstract

**Objectives:**

This study was conducted to report the course of an accidental release of chlorine gas that occurred in a factory in Gumi-si, South Korea, on March 5, 2013. We describe the analysis results of 2 patients hospitalized because of chlorine-induced acute health problems, as well as the clinical features of 209 non-hospitalized patients.

**Methods:**

We analyzed the medical records of the 2 hospitalized patients admitted to the hospital, as well as the medical records and self-report questionnaires of 209 non-hospitalized patients completed during outpatient treatment.

**Results:**

Immediately after the exposure, the 2 hospitalized patients developed acute asthma-like symptoms such as cough and dyspnea, and showed restrictive and combined pattern ventilatory defects on the pulmonary function test. The case 1 showed asthma-like symptoms over six months and diurnal variability in peak expiratory flow rate was 56.7%. In case 2, his FEV1 after treatment (93%) increased by 25% compared to initial FEV1 (68%). Both cases were diagnosed as chlorine-induced reactive airways dysfunction syndrome (RADS) on the basis of these clinical features. The most frequent chief complaints of the 209 non-hospitalized patients were headache (22.7%), followed by eye irritation (18.2%), nausea (11.2%), and sore throat (10.8%), with asymptomatic patients accounting for 36.5%. The multiple-response analysis of individual symptom revealed headache (42.4%) to be the most frequent symptom, followed by eye irritation (30.5%), sore throat (30.0%), cough (29.6%), nausea (27.6%), and dizziness (27.3%).

**Conclusions:**

The 2 patients hospitalized after exposure to chlorine gas at the leakage site showed a clinical course corresponding to RADS. All of the 209 non-hospitalized patients only complained of symptoms of the upper airways and mucous membrane irritation.

## Background

Chlorine is a yellow-green non-combustible gas with a pungent irritating odor. It exists as a gas at normal ambient temperature, has a higher density than air, and has mid-range hydrophilicity. It is widely used in industries and is one of the most commonly produced chemical substances worldwide. In particular, it is utilized as a reagent in the fabrication of solvents, pesticides, polymers, synthetic rubbers, refrigerants, and plastic, and as a bleach agent in the pulp and paper industry. It is also used as a disinfectant for purifying water [1],[2]. People are exposed to chlorine gas released by industries, either chronically by handling chloric materials or acutely through exposure to high-concentration chlorine because of accidents or careless handling. Chlorine exposure also occurs in daily life in many ways, for example, when using detergents or mixing disinfectants for domestic use [3].

Acute exposure to chlorine gas tends to cause mostly respiratory symptoms. Chlorine gas is partially soluble in water, and upon inhalation is often deposited on hygroscopic surfaces such as the eyes, nose, pharynx, and nasopharyngeal airways. Acute exposure to chlorine gas may initially cause eye and throat irritation. Such exposures can result in symptoms of acute airway obstruction, including wheezing, cough, chest tightness, and dyspnea. Clinical signs including hypoxemia, wheezes, rales, and abnormal chest radiographs may be present. The clinical expressions of chlorine gas inhalation include rhinitis, tracheobronchitis, airway hyperresponsiveness, reactive airways dysfunction syndrome (RADS), and bronchiolitis. More severely affected individuals may develop acute lung injury and acute respiratory distress syndrome [2],[3].

Accidental leakage of chlorine occurred on March 5, 2013, at 8:58 a.m., in a factory handling chlorine in Gumi-si, South Korea. The cause of the leak was assumed a ventilation error, which released chlorine gas to the work area, instead of absorbing it. Immediately after the accident, the workers at the leakage site and those in the adjacent factories as well as passers-by complained of respiratory symptoms and other health problems. Disaster mitigation measures were taken immediately; workers and the residents of the adjacent areas were evacuated 25 min after the accident, and the affected area was cordoned off to prevent further exposures. There were no fatalities due to the accident; however, many workers and residents experienced adverse health effects [4].

Incidents and accidents involving chlorine gas release are common worldwide. In developed countries, there have been occasional reports about the respiratory hazards of acute chlorine gas inhalation due to malfunction, leakage, or explosion in chlorine installations [5]-[7]. In the United States, for example, the Agency for Toxic Substances and Disease Registry performed a retrospective analysis on the public health consequences of acute chlorine releases in 16 states between 1993 and 2000, using the Hazardous Substances Emergency Events Surveillance system. The analysis revealed 865 reported incidents involving chlorine releases [7]. In South Korea, there have been reports of several cases of acute health problems due to exposure to chlorine during water purification processes [8],[9]; however, studies investigating the health effects of collective exposure to chlorine gas are rare. This study reports the course of an accidental release of chlorine gas that occurred in a factory in Gumi-si, South Korea, on March 5, 2013, and describes the results of the analysis of 2 hospitalized patients with chlorine-induced acute health problems as well as the clinical features of non-hospitalized patients.

## Materials and methods

Chlorine leakage was occurred at a chlorine processing company which is located in a national industrial complex in Gumi-si. On the day of the accident, liquefied chlorine gas stored in the basement of the factory was being filled into a tank on the ground floor. The pipe flow system stopped mid-operation because of an electric overload, and chlorine gas was released into the work area from the pipeline, instead of traveling up to the scrubber tower where it is supposed to be neutralized. The leakage volume was approximately 1 L by liquefied volume. When the 1 L was evaporated, the airborne volume was estimated approximately 400 L. The ambient chlorine gas measurement was done by industrial hygienist team of a university hospital. The local governmental agency for labor and employment had requested the measurement. The research team measured the chlorine concentration of the ambient air at the leakage site and in the adjacent areas 2 h after the accident. Three spots, each at a distance of approximately 100 m from the accident site, were chosen for measurement. Air specimens were collected for 6 hours by the area sampling using a low-volume flow sampler and quantitatively analyzed using ion chromatography [10].

The total number of patients treated for symptoms from the exposure to chlorine gas after this accident was 224; 2 hospitalized patients were admitted to the Department of pulmonology and 222 the non-hospitalized patients to the emergency department and the Department of Occupational and Environmental Medicine of the university hospital that is about 1 km away from the leakage site. The 2 hospitalized patients were analyzed by investigating their medical records. The clinical features of the non-hospitalized patients were analyzed using the questionnaire designed by the research team, as well as using their medical records. Of the 222 non-hospitalized patients, 209 were analyzed after excluding 13 patients who did not have questionnaire survey records.

The outpatients treated in the Department of Occupational and Environmental Medicine were asked to fill in the questionnaire after providing informed consent prior to treatment. The questionnaire items included basic demographic characteristics, their presence/absence at the leakage site at the time of the leakage, distance from the leakage site, duration of exposure, chief complaints, concomitant symptoms, medical history, and smoking history. For purposes of distance-related comparisons, 100 m was considered the reference distance. The subjects were asked to undergo a complete blood cell counts, hepatic function test, serum electrolyte test, urinalysis, and chest radiography. Patients with no or negligible symptoms and those who refused the tests were entirely or partially excluded from the test.

This study was approved by the Institutional Review Board of the Soonchunhyang University Gumi Hospital (SCHGM IRB 2013–12).

Data analyses were performed using the statistical package SPSS for Windows (version 14.0, USA). The distance-dependent comparison of symptoms was performed using the chi-squared test. The clinical laboratory test values were compared using the *t*-test.

## Results

### Case 1

 Sex/age: Male/38 years.

 Case description: The patient was driving near the leakage site. At a distance of 50 m from the leakage site, he opened the car window to smoke and perceived a strange odor. Upon exposure to chlorine gas, he experienced dyspnea and presented to the emergency department. The results of arterial blood gas analysis (ABGA) and chest radiography performed in the emergency department immediately after the accident did not reveal any abnormalities, and the patient was discharged on the same day upon the improvement of symptoms after conservative treatment. However, he was admitted to the pulmonology on the 2nd day post-exposure because of continuing cough and dyspnea.

 Medical history: No abnormalities.

 Familial history: No abnormalities.

 Personal history: He was a non-alcoholic and had been smoking about half a pack of cigarettes a day for 16 years. He had no asthmatic or allergy-related symptoms before the accident.

 Occupational history: He had been working as a delivery driver for about 15 years for a vending machine management company without any other particular occupational background.

 Physical examination findings: The vital signs measured at the time of admission to the emergency department were as follows: systolic/diastolic blood pressures, 130/80 mm Hg; pulse and respiration rates, 88 beats/min and 20 breaths/min, respectively; and body temperature, 37°C. Auscultation did not reveal rales or wheezing, except for coarse respiratory sounds. Neither fever symptoms nor skin rashes were observed.

 Laboratory findings: The ABGA performed immediately after the accident yielded the following values: pH, 7.37; PaCO_2_, 48.9 mm Hg; PaO_2_, 91.4 mm Hg; HCO_3_^−^, 27.7 mmol/L; and O_2_ saturation, 96.7%. In the pulmonary function test performed on the 2^nd^ day of admission, a mild restrictive pattern was observed with a forced vital capacity (FVC) of 66%, forced expiratory volume in 1 s (FEV1) of 83%, FEV1/FVC of 98%, and vital capacity of 66%. The diffusing capacity divided by the alveolar volume (DLco/VA) was reduced to 77% of the predicted value. The following values were recorded at the time of admission: (1) ABGA: pH, 7.42; PaCO_2_, 43.4 mm Hg; PaO_2_, 83.7 mm Hg; HCO_3_^−^, 27.4 mmol/L; O_2_ saturation, 96.5%. (2) Complete blood cell counts revealed hemoglobin level, 16.0 g/dL; hematocrit, 46.7%; white blood cells (WBC) count, 18,440/mm^3^ (neutrophils, 88.1%; lymphocytes, 6.6%); platelet count, 226,000/mm^3^. (3) Hepatic function test, serum electrolyte, serum glucose, and urinalysis demonstrated normal ranges. The chest radiograph did not reveal any abnormalities.

**Figure 1:**
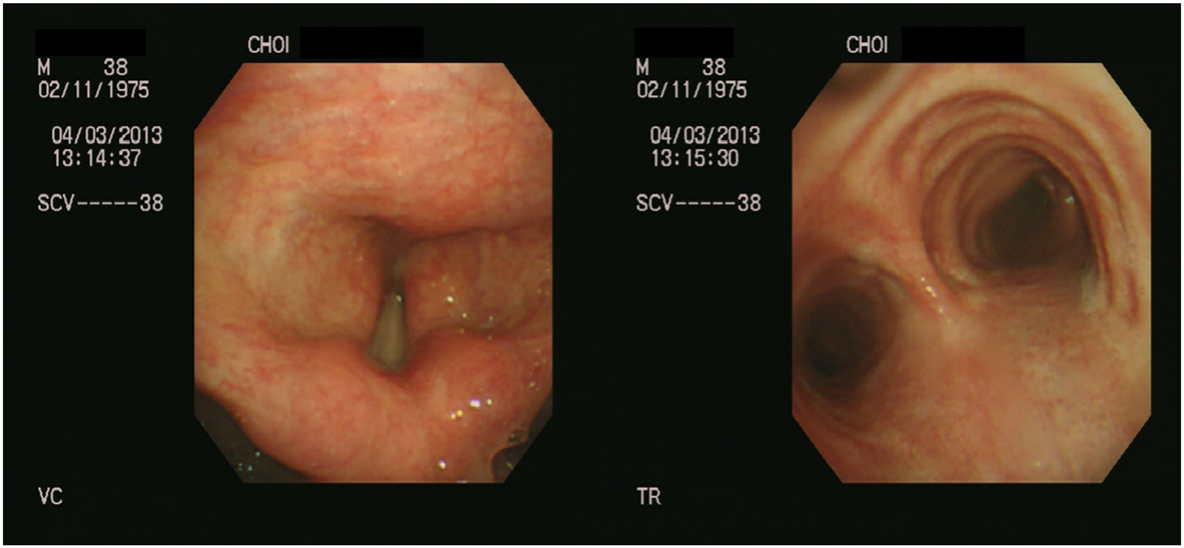
Bronchoscopic findings of case 1 showed no abnormality except mild hyperemic bronchus.

**Figure 2:**
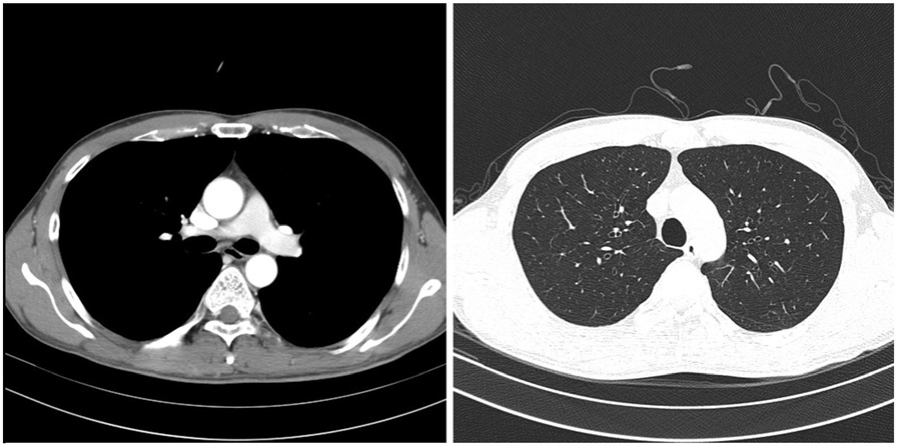
Chest CT findings of case 1 show no abnormality.

 Treatment and clinical outcome: The hospital treatment included empirical broad-spectrum antibiotics and administration of a bronchodilator. On the 2^nd^ day of hospitalization, coughing, sputum, and shortness of breath reduced, and vital symptoms became stable. The methacholine challenge test performed on the 6^th^ day of hospitalization showed a negative result. The patient was discharged on the 6^th^ day upon the improvement of symptoms. On March 18, the patient was re-admitted to the department of pulmonology because of continuing cough and dyspnea. The pulmonary function test performed at March 18 revealed FVC, FEV1, and FEV1/FVC values of 77%, 96%, and 97%, respectively. The patient was hospitalized for one month. During the hospitalization, chest computerized tomography (CT) scan and bronchoscopy were performed, they did not reveal any abnormalities (Figures 1 and 2). From March 19 through April 15, the peak expiratory flow rate was measured 14 times. The minimum value was 240 ml and the maximum value was 430 ml, diurnal variability in peak expiratory flow rate was 56.7%. After discharge, treatment included inhaled corticosteroid and administration of bronchodilator for six months and the respiratory symptoms including cough, dyspnea virtually vanished.

### Case 2

 Sex/age: Male/35 years.

 Case description: The patient is a worker in the factory. He was exposed to chlorine gas while he was charging the chlorine gas stored in the basement to the tank on the ground floor. He was not wearing any safety devices. He perceived a very strong smell similar to that of a swimming pool disinfectant at 09:06 a.m., 8 min after the leakage. He experienced eye and mucous membrane irritations, and he cut the valve releasing the chlorine gas. The amount of released chlorine was approximately 1 L. He presented to the emergency department with symptoms of cough, chest discomfort, and palpitation.

 Medical history: No abnormalities.

 Familial history: No abnormalities.

 Personal history: He had been consuming a bottle (375 mL) of soju (distilled beverage with approximately 20% alcohol content by volume) 5 times a week and smoking a pack of cigarettes a day for 15 years. He had no asthma or allergy-related symptoms before the manifestation of the current symptoms.

 Occupational history: He had been working in the concerned factory, assigned to transporting, storing, and charging chlorine gas, for about 5 years without any other particular occupational background.

 Physical examination findings: The vital signs measured at the time of admission to the emergency department were as follows: systolic/diastolic blood pressures, 100/70 mm Hg; pulse and respiration rates, 80 beats/min and 28 breaths/min, respectively; and body temperature, 36.5°C. Auscultation did not reveal any rales or wheezing, except for coarse respiratory sounds. Neither fever symptoms nor skin rashes were observed.

 Laboratory findings: The initial ABGA performed immediately after the accident (09:23 a.m.) yielded the following values: pH, 7.28; PaCO_2_, 33.5 mm Hg; PaO_2_, 68.4 mm Hg; HCO_3_^−^, 15.5 mmol/L; and O_2_ saturation, 93%, which indicated metabolic acidosis and hypoxemia. Complete blood cell counts yielded the following values: hemoglobin level, 15.6 g/dL; hematocrit, 46.7%; WBC count, 7300/mm^3^ (neutrophils, 49.3%; lymphocytes, 42.6%); and platelet count, 326,000/mm^3^. A hepatic function test measured the values of serum aspartate transaminase, serum alanine transaminase, and postprandial glucose as 29 IU/L, 19 IU/L, and 149 mg/dL, respectively. Serum electrolyte, serum glucose, and urinalysis revealed normal ranges.

 Treatment and clinical outcome: The chest radiograph taken on admission did not reveal any abnormalities; however, his SpO_2_ (arterial oxygen saturation measured by pulse oximetry) was 83%. The 2^nd^ ABGA performed at 10:04 a.m. showed values of pH, 7.34; PaCO_2_, 42.6 mm Hg; PaO_2_, 50.1 mm Hg; HCO_3_^−^, 22.9 mmol/L; and O_2_ saturation, 84.3%, indicating metabolic acidosis and hypoxia. Therefore, 100% O_2_ was administered at the rate of 10 L/min. For the treatment of metabolic acidosis, sodium bicarbonate dissolved in saline solution was intravenously injected. The 3^rd^ ABGA performed at 11:50 a.m. showed improved values of pH, 7.43; PaCO_2_, 39.2 mm Hg; PaO_2_, 96.7 mm Hg; HCO_3_^−^_,_ 25.5 mmol/L; and O_2_ saturation, 97.5%. The patient was moved to the intensive care unit and given inhalation therapy of salbuterol, budesonide, and ipratropium 4 times a day, along with intravenous treatment of ascorbic acid, methyl predinosolne, and third-generation cephalosporin antibiotics. O_2_ (100%) was supplied at the rate of 10 L/min, and SpO_2_ was maintained at 95%. Auscultation revealed rales in both lungs, but the chest radiograph taken did not reveal any abnormalities. The pulmonary function test performed on the 2^nd^ day of hospitalization showed the following values: FVC, 77%; FEV1, 68%; FEV1/FVC, 71%; forced expiratory flow rate (FEF; 25–75%), 42%; and DLco, 71%. The ABGA performed on the 5^th^ day of hospitalization after 30-min discontinuation of oxygen inhalation showed the following values: pH, 7.37; PaCO_2_, 45.4 mm Hg; PaO_2_, 63.3 mm Hg; HCO_3_^−^, 24.1 mmol/L, and O_2_ saturation, 91.4%. The pulmonary function test performed on the 5^th^ day yielded values of FVC, 86%; FEV1, 82%; FEV1/FVC, 75%; and FEF, (25–75%), 58%, and the patient had a mild cough. The same test performed 7^th^ days yielded values of FVC, 94%; FEV1, 93%; FEV1/FVC, 79%; and FEF, (25–75%), 73%. The patient refused further treatment and follow-up examinations; he was discharged at 7^th^ admission day.

### Clnical features of non-hospitalized patients

The general characteristics of the non-hospitalized patients affected by chlorine exposure are as follows: male patients were dominant, 73.4%; in the age distribution, patients in their 30s (37.4%) were the most frequent, followed by ≤29 year olds (29.6%), 40–49 year olds (24.6%), and ≥50 year olds (8.4%); by occupation, most of the patients were workers (54.6%), followed by residents in the adjacent area (28.6%), others (15.3%), and police officers and firefighters (1.5%); smokers accounted for 27.6%; most patients (70.0%) presented to the hospital within 24 h of exposure, and 18.7% presented within 2 days; those who were within 100 m distance accounted for 35.9%, followed by 100–500 m (52.7%) and ≥500 m (11.4%); the duration of exposure was 1–2 h (29.1%), 4–6 h (23.6%), and ≤1 h (22.7%) (Table 1).

**Table 1:** Characteristics of non-hospitalized patients with respect to the distance from the accident spot

	**Within 100 m**		**Beyond 100 m**		**Total**		p-value*
	**n**	**%**	**n**	**%**	**n**	**%**	
**Sex**							0.639
Male	55	75.3	94	72.3	149	73.4	
Female	18	24.7	36	27.7	54	26.6	
**Age**							0.095
<29 years	26	35.6	34	26.2	60	29.6	
30–39 years	25	34.2	51	39.2	76	37.4	
40–49 years	20	27.4	30	23.1	50	24.6	
>50 years	2	2.7	15	11.5	17	8.4	
**Exposure type**							0.016
Workers	43	58.9	68	52.3	111	54.7	
Adjacent residents	25	34.2	33	25.4	58	28.6	
Firefighters and officer	1	1.4	2	1.5	3	1.5	
Others	4	5.5	27	20.8	31	15.3	
**Smoking**							0.020
Non-smoking	60	82.2	87	66.9	147	72.4	
Smoking	13	17.8	43	33.1	56	27.6	
**Time of 1**^**st**^**hospital visit**							0.099
1^st^ day	42	57.5	100	76.9	142	70.0	
2^nd^ day	24	32.9	14	10.8	38	18.7	
After 3^rd^ day	7	9.6	16	12.3	23	11.3	
**Period of stay**							<0.001
Within 1 h	22	30.1	22	18.4	44	22.7	
1–2 h	32	43.8	27	20.8	59	29.1	
2–4 h	4	5.5	20	15.4	24	11.8	
4–6 h	5	6.8	43	33.1	48	23.6	
>6 h	10	13.7	16	12.3	26	12.8	

*Chi-squared test.

For comparison purposes, we divided the subjects into those within 100 m and those beyond 100 m, depending on the distance from the leakage site. There were no sex-dependent differences between within 100 m and beyond 100 m group. The age distribution also showed no differences between 2 groups. In terms of occupational exposure, the percentage of workers to residents in the within 100 m group was 58.9% vs. 34.2%, and that of the beyond 100 m group was 52.3% vs. 25.4%. There was a significant difference in the percentage of smokers by distance; 17.8% were smokers in the within 100 m group and 33.1% in the beyond 100 m group. While the patients who presented to the hospital on the 1^st^ and 2^nd^ day of the accident were 57.5% and 32.9%, respectively, in the within 100 m group, the number in the beyond 100 m group was 76.9% and 10.8%, respectively. As for the duration of exposure of the within 100 m group, subjects who reported in <1 h accounted for 30.1%, 1–2 h 43.8%, and 2–4 h 5.5%; for the beyond 100 m group, the values were 18.4%, 20.8%, and 15.4%, respectively, presenting significant differences (p < 0.001) (Table 1).

The most frequent chief complaints of the patients were headache (22.7%), followed by eye irritation (18.2%), nausea (11.3%), and sore throat (10.8%), with 36.5% not showing any chief complaints. The comparison of frequency of chief complaints by distance between within and beyond the 100 m group was 31.5% vs. 10.8% for eye irritation, with the within 100 m group showing a significantly high frequency (p < 0.001). The proportion for patients with no symptoms was 26.0% vs. 42.3%, indicating that patients who were farther away from the accident spot more had no symptoms as a chief complaints (p = 0.021). No distance-dependent differences were observed for other symptoms (Table 2).

**Table 2:** Chief complaints of non-hospitalized patients with respect to the distance from the accident spot

**Chief complaint**	**Within 100 m**		**Beyond 100 m**		**Total**		p-value^*^
	**n**	**%**	**n**	**%**	**n**	**%**	
Headache	20	27.4	26	20.0	46	22.7	0.227
Eye irritation	23	31.5	14	10.8	37	18.2	<0.001
Nausea	10	13.7	13	10.0	23	11.3	0.425
Sore throat	10	13.7	12	9.2	22	10.8	0.326
Chest pain	10	13.7	9	6.9	19	9.4	0.112
Dizziness	7	9.6	5	3.8	12	5.9	0.096
Cough	1	1.4	9	6.9	10	4.9	0.079
Other	10	13.7	25	19.2	35	17.2	0.317
No symptom	19	26.0	55	42.3	74	36.5	0.021
Total	73	100.0	130	100.0	203	100.0	

*Chi-squared test.

The multiple-response analysis of individual symptoms revealed headache (42.4%) to be the most frequent symptom, followed by eye irritation (30.5%), sore throat (30.0%), cough (29.6%), nausea (27.6%), and dizziness (27.3%). Responses that showed differences between the within 100 m and beyond 100 m groups were shortness of breath (30.1% vs. 9.2%; p < 0.001), sore throat (37.0% vs. 26.2%; p = 0.016), eye irritation (42.5% vs. 23.8%; p = 0.006), itching (17.8% vs. 6.9%; p = 0.017), dizziness (37.0% vs. 23.1%; p = 0.020), anxiety (21.9% vs. 5.4%; p < 0.001), general weakness (16.4% vs. 6.2%; p = 0.018), and fatigue (35.6% vs. 16.9%; p = 0.003), with the within 100 m group demonstrating significantly higher frequencies of these complaints. On the other hand, no significant distance-dependent differences were observed in symptoms such as cough, sputum, nasal pain, dental pain, eye redness, blurred vision, headache, nausea, and chest discomfort (Table 3).

**Table 3:** Comparison of symptom complaints with respect to distance from the accident spot

**Chief complaint**	**Within 100 m**		**Within 100 m**		**Total**		p-value^*^
	**n**	**%**	**n**	**%**	**n**	**%**	
**Lung**							
Cough	19	26.0	41	31.5	60	29.6	0.409
Shortness of breath	22	30.1	12	9.2	34	16.7	0.000
Sputum	11	15.1	22	16.9	33	16.3	0.731
**Nose and neck**							
Sore throat	27	37.0	34	26.2	61	30.0	0.016
Nasal pain	9	12.3	13	10.0	22	10.8	0.608
Dental pain	24	32.9	35	26.9	59	29.1	0.370
**Eye**							
Eye pain	31	42.5	31	23.8	62	30.5	0.006
Eye redness	8	11.0	12	9.2	20	9.9	0.692
Blurred vision	9	12.3	11	8.5	20	9.9	0.375
**Skin**							
Itching	13	17.8	9	6.9	22	10.8	0.017
**Nerve**							
Headache	36	49.3	50	38.5	86	42.4	0.133
Dizziness	27	37.0	30	23.1	57	27.3	0.020
**Stomach**							
Nausea	22	30.1	34	26.2	56	27.6	0.542
**Heart**							
Chest discomfort	21	28.8	28	21.5	49	24.1	0.248
**Psychological**							
Anxiety	16	21.9	7	5.4	23	11.3	<0.001
**Other**							
General weakness	12	16.4	8	6.2	20	9.9	0.018
Fatigue	26	35.6	22	16.9	48	23.6	0.003
**Total**	73	100.0	130	100.0	203	100.0	

*Chi-squared test.

**Figure 3:**
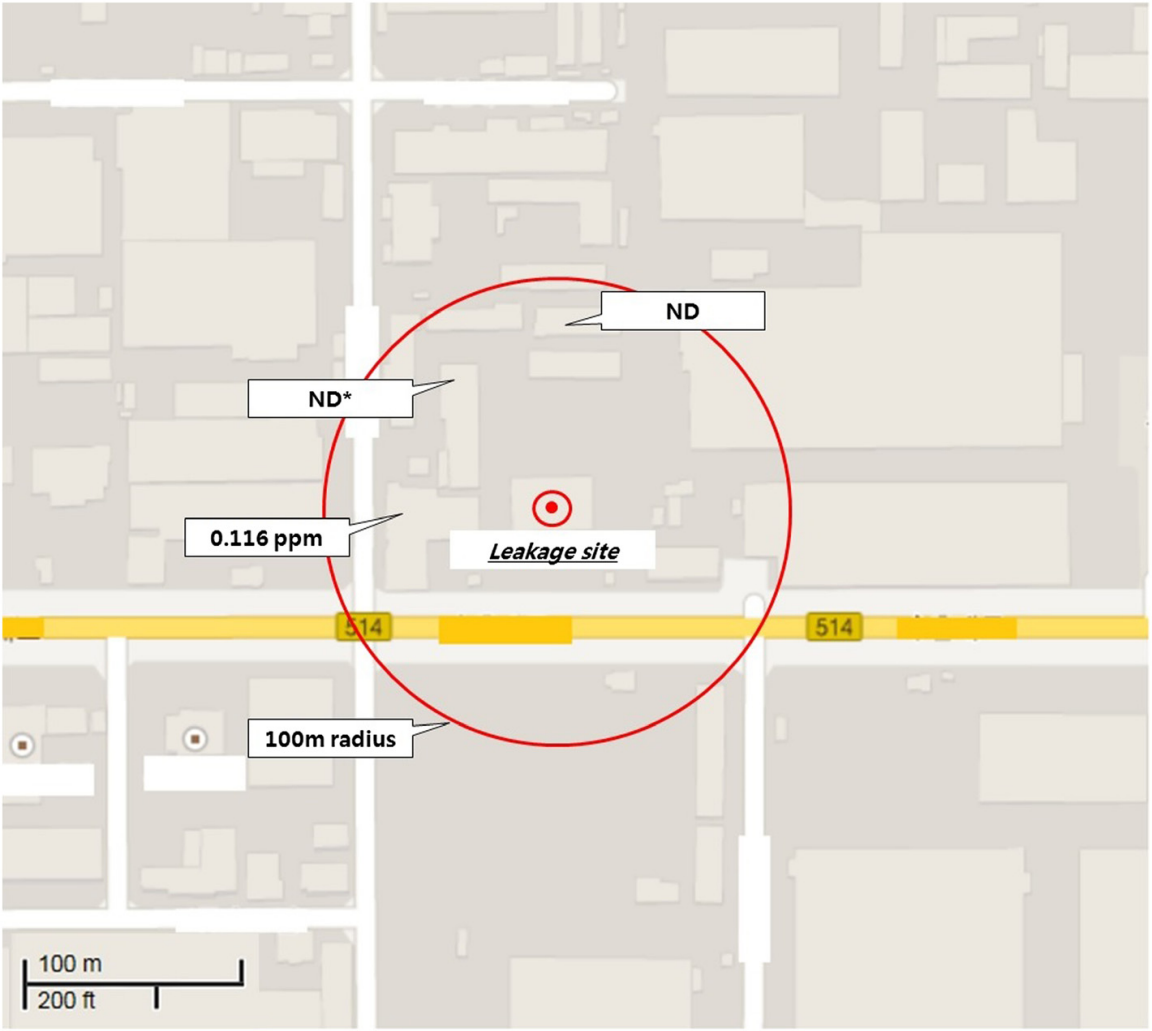
**Environmental chlorine gas concentration 2 h after the accident.** *ND, Non-detectable.

The chlorine gas concentration of the ambient air was measured at three spots. 0.116 ppm of chlorine was measured at one spot and no chlorine was detected at the other 2 spots (Figure 3).

Clinical laboratory tests were performed on 188 subjects out of the 209 outpatients. Among the hematological and clinicochemical profiles, only total cholesterol level showed a difference between the within and beyond 100 m groups (182.8 ± 31.9 mg/dL vs. 196.1 ± 35.5 mg/dL; p = 0.014), showing that the total cholesterol level was inversely proportional to the distance (Table 4).

**Table 4:** Comparison of laboratory results by distance from the accident spot

	**Within 100 m**	**Beyond 100 m**	**p-value**
	**Mean ± SD**	**Mean ± SD**	
**Complete blood cell count**			
WBC (×10^3^/mm^3^)	7.13 ± 1.78	7.12 ± 1.89	0.989
RBC (×10^6^/mm^3^)	4.80 ± 0.43	4.83 ± 0.56	0.655
Hemoglobin (g/dL)	14.7 ± 1.36	14.9 ± 1.40	0.406
Hematocrit (%)	43.5 ± 3.56	44.0 ± 3.52	0.359
Platelet (×10^3^/mm^3^)	222.1 ± 42.4	233.5 ± 48.9	0.114
**Serum electrolyte**			
Calcium (mg/dL)	9.16 ± 0.28	9.17 ± 0.32	0.784
Phosphorus (mg/dL)	3.66 ± 0.52	3.71 ± 0.52	0.579
Sodium (mEq/dL)	141.9 ± 1.58	141.9 ± 1.56	0.835
Potassium (mEq/dL)	4.01 ± 0.23	4.01 ± 0.31	0.913
Chloride (mEq/dL)	101.3 ± 1.16	102.6 ± 1.19	0.246
**Serum chemistry**			
AST^†^ (IU/L)	26.1 ± 26.7	24.5 ± 22.2	0.667
ALT^‡^ (IU/L)	28.1 ± 21.5	27.2 ± 18.8	0.793
r-GTP^§^ (IU/L)	34.7 ± 44.9	29.4 ± 24.6	0.310
ALP^║^ (IU/L)	153.1 ± 33.9	157.1 ± 44.7	0.790
Glucose (mg/dL)	98.5 ± 16.1	102.6 ± 26.9	0.263
Total cholesterol (mg/dL)	182.8 ± 31.9	196.1 ± 35.5	0.014
Blood urea nitrogen (mg/dL)	13.1 ± 3.03	13.4 ± 3.08	0.505
Creatinine (mg/dL)	0.89 ± 0.23	0.90 ± 0.16	0.695
Uric acid (mg/dL)	5.43 ± 1.45	5.50 ± 1.59	0.759
**Abnormal chest radiograph**	0/73	0/129	

^*^Independent *t*-test.

^†^Aspartate transaminase.

^‡^Alanine transaminase.

^§^Gamma-glutamyl transpeptidase.

^║^Alkaline phosphatase.

Chest radiographs taken from 202 subjects did not yield any findings that indicated acute respiratory disorders.

## Discussion

Chlorine poisoning can occur in swimming pools when accidents with water purification systems occur, during military exposures, after accidents during the transport of the chemical, upon industrial exposure, and with misuse of domestic cleaners [1],[2]. Chlorine poisoning can be categorized into 2 types: poisoning from exposure to chlorine itself, and from chlorine released as a by-product from the reaction between materials containing hypochloric acid (HOCl) and chloride [2],[11]. The situation described in this article belongs to the 1^st^ category. The American Conference of Governmental Industrial Hygienists specifies the permissible time-weighted average of chlorine exposure in ambient air at 0.5 ppm, and the short-term exposure limit at 1 ppm. The time-weighted average and short-term exposure limit permissible in South Korea are 1 and 3 ppm, respectively. The smell of chlorine gas is perceivable by humans at an estimated ambient concentration of 0.2 ppm, eye and mucous membrane irritations may occur at 3–15 ppm, and an exposure of 5–10 min at 15–150 ppm concentrations is enough to induce chronic respiratory disorders. It is also estimated that an exposure >30 min at 400–500 ppm concentrations is lethal to half of the healthy persons exposed [12]. The estimated chlorine exposure levels in our case were ≥15 ppm for hospitalized patients and 3–15 ppm for non-hospitalized patients.

The 2 hospitalized patients, who had no medical history of respiratory disorders, showed asthma-like symptoms such as cough and shortness of breath within 24 h of acute chlorine inhalation exposure, where case 1 and 2 showed restrictive pattern ventilatory defects and combined pattern ventilatory defects, respectively, in the pulmonary function test. Although metacholine challenge test was negative in case 1, the case 1 showed asthma-like symptoms over six months and diurnal variability in peak expiratory flow rate was 56.7%. When diurnal variability in peak expiratory flow rate exceeds 20%, it is possible to diagnose as asthma [13]. In case 2, despite of the medical staff’s recommendation, he refused further accurate evaluation. Therefore we could not evaluate for bronchial hyperresponsiveness. But his FEV1 after treatment (93%) increased by 25% compared to initial FEV1(68%). If FEV1 increased after treatment, it indicates the possibility of asthma [13]. Taking these clinical features into consideration, the investigators diagnosed both cases as chlorine-induced RADS [14],[15]. RADS is a type of irritant-induced asthma without a latency period [16],[17]. RADS was defined by Brooks et al. in 1985 [18] as having the following diagnostic characteristics: (1) a documented absence of preceding respiratory complaints; (2) onset of symptoms after a single exposure incident or accident; (3) exposure to a gas, smoke, fume, or vapor with irritant properties present in very high concentrations; (4) onset of symptoms within 24 h after the exposure, with persistence of symptoms for at least 3 months; (5) symptoms simulating asthma with cough, wheeze, and dyspnea; (6) presence of airflow obstruction on pulmonary function tests and/or the presence of non-specific bronchial hyperresponsiveness; and (7) with other pulmonary diseases ruled out. Later, Bardana proposed the diagnostic criteria for RADS in 1995, which include a requirement for histopathological analysis showing minimal lymphocytic inflammation without eosinophilia [19]. However, there is no “gold standard” objective test for this diagnosis. Therefore, accurate diagnosis depends on the patient’s history. The clinical course of RADS is predicted according to the irritant substances and exposure duration. Previous papers identified chloride as the most common irritant, followed by toluene diisocyanate and nitrogen oxide [20].

While the exact pathogenesis of chlorine gas in inducing RADS has not been elucidated yet, there are some salient points advanced by the claim that inhalation exposure to high-concentration irritants causes epithelial damage of bronchial mucosa, which leads to threshold shift of receptors or increase in epithelial permeability, therefore, inducing bronchial hyperresponsiveness [21],[22]. In previous studies, ABGA performed in patients with chlorine-induced RADS revealed metabolic acidosis and hypoxemia [23]-[25], which is consistent with the clinical findings of our 2 inpatient cases. As chlorine-induced RADS usually manifests as airway inflammation symptoms, no abnormalities were found on radiography, as was the case with our 2 inpatients. The change in pulmonary function immediately after an inhalation exposure to chlorine can be manifested as restrictive and combined pattern ventilatory defects. In most cases, improvements are observed over time, after a reversible course [24],[25]. However, there are also cases of exacerbated non-specific airway hyperresponsiveness. The impaired pulmonary function manifested as restrictive and combined pattern ventilatory defects by case 1 and 2, respectively, of this study also improved over time. But case 1 did not show non-specific bronchial hyperresponsiveness, indicated by a negative result in the methacholine challenge test performed on the 6^th^ day of hospitalization.

The main treatment methods for RADS are intravenous steroid injection and bronchodilator inhalation therapy for bronchial extension to treat ventilatory defects, which are well-established therapies for RADS, as verified by many previous studies that reported that patients treated with steroid and bronchodilator can more rapidly counteract pulmonary function impairment and histological degradation [26]-[29]. Steroids and bronchodilators were also used to treat the patients in our study, and broad-spectrum antibiotics were used to prevent secondary respiratory complications. One study also found that rats treated with antioxidants after chlorine exposure showed a 4-fold lower mortality compared with the control group [30]. Relying on this finding, we administered ascorbic acid to patient 2 during his hospitalization. Rapid and full recovery from chlorine gas-induced RADS is the most likely outcome, and many papers have reported that pulmonary function generally recovers to the normal level even after an exposure to high-concentration chlorine [31]-[33]. The patients in this study were also discharged after their rapid recoveries. On the other hand, there is also a case report in which an asthma case discovered in a follow-up after 1 year was successfully treated [9], and another report in which workers exposed to chlorine during pulp-mill processes developed respiratory symptoms and bronchial hyperresponsiveness up to 18–24 months after the exposure [32]. This suggests the need for long-term follow-up for our cases as well. Considering the smoking factor in conjunction with RADS, Hassan et al. verified slow recoveries in 6 patients with dyspnea while investigating the severity of airway obstruction and clinical features of 18 patients acutely exposed to chlorine gas, and explained it to be associated with their smoking and lung disease histories [33]. Both hospitalized patients in our study were smokers. Although smoking may be assumed to have influenced their clinical course, we did not conduct relevant analyses; further research may be needed to address this issue.

On the other hand, none of the 209 non-hospitalized patients showed any abnormalities in clinical laboratory tests, and we could not find any symptoms indicative of acute target organ damage. They complained of symptoms limited to upper airway and mucous membrane irritations. Their most frequent chief complaints was headache (22.7%), followed by eye irritation (18.2%), nausea (11.3%), and sore throat (10.8%). According to the frequency of individual symptoms, headache accounted for 42.4%, followed by eye irritation (30.5%), sore throat (30.0%), cough (29.6%), nausea (27.6%), and dizziness (27.3%). These symptoms are considered attributable to upper airway and mucous membrane irritation induced by exposure to low-concentration chlorine.

Chlorine is 1.5 to 2 times heavier than air; therefore, instead of rapidly rising in the atmosphere and being diluted, it mostly remains at the ground level near the leak site in high concentrations and spreads along the ground [2]. In the factory concerned, the premises were within the 100 m mark from the leakage site, and most workers directly exposed to chlorine were within this distance. Considering this particularity, we took 100 m as the dividing mark for comparison of individual characteristics in estimating the severity of exposure. Many non-hospitalized patients who complained of eye irritation as chief complaints were near the leakage site. Individual symptoms, such as shortness of breath, sore throat, itching, dizziness, anxiety, general weakness, and fatigue, were also reported more frequently in proportion to the vicinity to the leakage site. This is assumed to be due to the higher chlorine concentration in proportion to the nearness to the leak site, thus evoking more severe symptoms of respiratory and mucous membrane.

There have been many case reports on acute health effects induced by chlorine exposure, but they have rarely been followed up by studies on the long-term effects on larger population of the affected communities after those massive accidental releases. One exemplary case report in this regard is a study dealing with a chlorine release accident that occurred in Graniteville, South Carolina, the United States, in January 2005 [34]. A rail derailment and subsequent crash of a freight train carrying liquefied chlorine released about 42–60 tons of chlorine gas in the premises of a textile plant. The chlorine gas thus released formed a cloud of dense gas and spread across the adjacent textile mill and the surrounding areas. Eight people died before reaching the hospital, and a total of 597 people presented to health-care facilities. Of them, 71 patients were hospitalized for acute health effects; 1 of these patients died. The analyses of the hospitalized patients revealed that all patients presented chest discomfort (31%), sore throat (15%), gastrointestinal symptoms (14%), eye irritation (13%), and dermatologic irritation (1%). These symptoms coincide with those presented in this study in which the non-hospitalized patients complained of headache, cough, sore throat, eye pain, nausea, dizziness, and chest comfort in decreasing order of frequency. A study performed in India analyzed the symptoms of 64 patients who presented to the hospital after exposure to chlorine gas while disinfecting a public bathhouse [35]. They complained of dyspnea and chest discomfort (100%), followed by cough (97%), eye irritation (88%), and runny nose (78%), similar to the findings of our study. Unlike these studies, however, headache was the most frequent complaint in our study; this may be attributable to the neurological stimulation evoked by the irritating odor of chlorine gas perceivable at low concentrations of <1 ppm [1],[3].

It is difficult to objectively estimate the exposure level of subjects in studies investigating environmental calamities caused by accidental chemical substance leakage. Although we measured the airborne concentrations of chlorine gas released across the community concerned 2 h after the accidental leakage, this was not enough to quantitatively estimate the individual exposure levels of the subjects. Officially confirmed exposure data was very important to describing health effects. In unexpected community environmental accident, timely environmental exposure monitoring is essential to prevent and evaluate health effects of victims. In a previous study investigating the accidental release of hydrogen fluoride that occurred in Gumi-si in September 2012 [36], patients’ individual exposure levels of were not known, and the distance from the leakage site was used as a proxy marker. The distance from the leakage site was also used in our study as an indicator indirectly reflecting the subjects’ exposure levels. There were many other factors that influences the exposure level of individual victims, such as wind direction, exact location of victims. But, we could not collect data about exact location of individual patient at the time of exposure except distance from accident site. If the patient was working in a space with all closed windows or doors, he would be minimally affected by the chlorine exposure, even though he was within 100 meters radius.

Another limitation of this study was that we could not estimate the total number of exposed victims. We could not find any formal accident report of government or other authorities which contain information about in total how many people were exposed. Additionally, the leakage site was located just aside to heavy traffic road and surrounded by many buildings, it was almost impossible to apprehend all exposed workers.

This study also had limited evaluation of the health effects on the non-hospitalized patients because of the lack of pulmonary function and bronchial hyperresponsiveness tests. In the 2 hospitalized patients, the limitations might be that no histopathological study was conducted for a more accurate diagnosis of RADS. Additionally, bronchial hyperresponsiveness was examined only in case 1.

According to a follow-up study conducted on 279 residents of adjacent areas 5 months after the accident where chlorine gas was released in Graniteville [37], 76 of 94 subjects included in the final analysis complained of chronic symptoms associated with chlorine exposure, and 47 were being treated in health-care institutions. Moreover, 44 subjects showed positive results of post-traumatic stress disorder in the questionnaire survey. The presence of residual symptoms in a large proportion of subjects even after several months suggests the necessity for follow-up observations for the subjects exposed to chlorine gas in the present study as well.

## Conclusions

The 2 patients hospitalized because of accidental chlorine gas release in Gumi-si showed clinical progressions corresponding to RADS. All 209 non-hospitalized patients complained of upper airway and mucous membrane irritation symptoms only and showed no abnormalities in clinical laboratory tests. Patients who were closer to the accident spot more frequently complained of symptoms such as shortness of breath, sore throat, eye pain, itching, dizziness, anxiety, general weakness, and fatigue. We consider it necessary to conduct a follow-up study to determine the long-term health effects of acute chlorine gas exposure.

## Consent

Written informed consent was obtained from the patient for the publication of thisreport and any accompanying images.

## Competing interests

The authors declare that they have no competing interests.

## Authors’ contributions

All authors had access to the data and played a role in writing the manuscript. JAK conceived and designed the study. SYY and SYC were involved in writing the manuscript. JHY and GIL performed the data collection. JSK and HSK performed the statistical analysis and the interpretation of data. JSK critically revised the manuscript. All authors read and approved the final manuscript.
